# Complete Genome Sequence of a Novel Coronavirus (SARS-CoV-2) Isolate from Bangladesh

**DOI:** 10.1128/MRA.00568-20

**Published:** 2020-06-11

**Authors:** Senjuti Saha, Roly Malaker, Mohammad Saiful Islam Sajib, Md Hasanuzzaman, Hafizur Rahman, Zabed B. Ahmed, Mohammad Shahidul Islam, Maksuda Islam, Yogesh Hooda, Vida Ahyong, Manu Vanaerschot, Joshua Batson, Samantha Hao, Jack Kamm, Amy Kistler, Cristina M. Tato, Joseph L. DeRisi, Samir K. Saha

**Affiliations:** a Child Health Research Foundation, Dhaka, Bangladesh; b MRC Laboratory of Molecular Biology, Cambridge, United Kingdom; c Chan Zuckerberg Biohub, San Francisco, California, USA; d Department of Biochemistry and Biophysics, University of California, San Francisco, San Francisco, California, USA; e Department of Microbiology, Dhaka Shishu Hospital, Bangladesh Institute of Child Health, Dhaka, Bangladesh; DOE Joint Genome Institute

## Abstract

The complete genome sequence of a novel coronavirus (severe acute respiratory syndrome coronavirus 2 [SARS-CoV-2]) isolate obtained from a nasopharyngeal swab from a patient with COVID-19 in Bangladesh is reported.

## ANNOUNCEMENT

Coronavirus disease 2019 (COVID-19) is an infectious disease caused by severe acute respiratory syndrome coronavirus 2 (SARS-CoV-2), which belongs to the family *Coronaviridae* and the genus *Betacoronavirus*. In Bangladesh, the first three cases were detected on 8 March 2020. By 14 May 2020, more than 18,000 cases and 280 deaths had been reported in the country. The Child Health Research Foundation (CHRF) has been a testing center for COVID-19 since 29 March 2020 and uses quantitative PCR (qPCR)-based SARS-CoV-2 detection in nasopharyngeal or oropharyngeal swab samples (1). Here, we report the complete sequence of a SARS-CoV-2 isolate from a patient who tested positive in a qPCR test.

All protocols were approved by the National Research Ethics Committee, Bangladesh Medical Research Council, and the ethical review board of the Bangladesh Institute of Child Health. Samples from suspected COVID-19 patients were collected for clinical care and diagnostic testing at the discretion of the attending health care providers and were received and tested at the CHRF with the permission of the Director General Health Services, Government of Bangladesh.

For this study, a nasopharyngeal specimen from a symptomatic patient with COVID-19 was collected on 18 April 2020. The specimen was tested for SARS-CoV-2 using qPCR at the CHRF and had a low cycle threshold value (*C_T_* < 15). Extraction of the viral nucleic acid from the nasopharyngeal specimen was performed using the Quick-RNA/DNA microprep extraction kit (product no. D7005; Zymo) according to the manufacturer’s protocol. cDNA was converted to Illumina libraries using the NEBNext Ultra II RNA library preparation kit (product no. E7770; NEB) according to the manufacturer’s protocol. Targeted enrichment of the SARS-CoV-2 sequence using 73 tiling primers at a 20:1 molar ratio of random primers to tiled primers was adapted from viral genome recovery methods described previously (2, 3). External RNA Controls Consortium Collection spike-in control mix (product no. 4456740; Thermo Fisher Scientific) was used as a marker of potential library preparation errors and for input RNA mass calculation. The library was sequenced on an Illumina iSeq100 sequencer using 150-nucleotide paired-end sequencing present at CHRF.

The library generated 15,376,000 reads, of which 11,747,398 passed the default Illumina quality filter in BaseSpace. Raw fastq files were uploaded to the IDSeq portal for host subtraction (4). SARS-CoV-2 reads were recovered by mapping the raw reads against the reference sequence (GenBank accession no. MN908947.3) using minimap2 (v2.17) (5, 6) and filtering the reads that mapped against a database of human and other viral genomes using Kraken2 (v2.0.8_beta) (7). The surviving reads were adapter trimmed with Trim Galore and remapped to the same reference using minimap2. Enrichment primers were trimmed with iVar (v1.2), and the consensus genome was called using iVar (8, 9). The full pipeline is available online (https://github.com/czbiohub/sc2-msspe-bioinfo).

**FIG 1:**
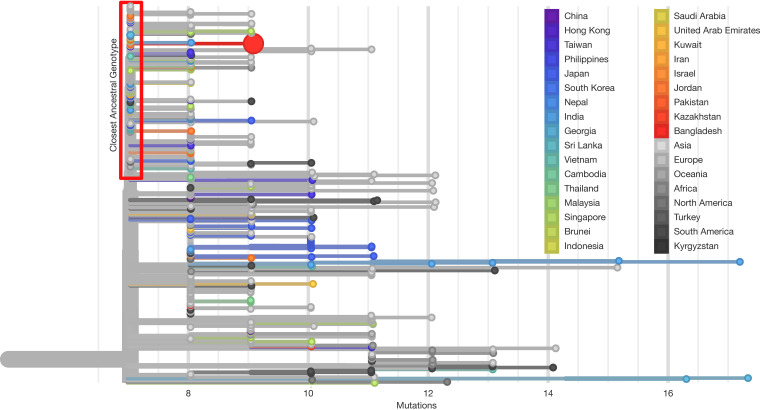
Phylogenetic tree of SARS-CoV-2 in the neighborhood of the first genome from Bangladesh. The *x* axis represents the number of mutations from the Wuhan strain (GenBank accession no. MN908947.3). The large red circle represents the position of CHRF_nCoV19_0001. Its closest ancestral genotype includes sequences from North America, Europe, the Middle East, East Asia, and Southeast Asia. The figure was rendered using Nextstrain (nextstrain.org).

The complete genome of the Bangladeshi SARS-CoV-2 strain (CHRF_nCoV19_0001) has 29,903 bp, with an average coverage of over 3,000×; no indels were detected, and the GC content was 38%. The sample was uploaded to the Global Initiative on Sharing All Influenza Data (GISAID) database (10) on 12 May 2020. The genome was subsequently contextualized among 5,265 other genomes available in the GISAID database in the Nextstrain Asia build of 15 May 2020 (www.nextstrain.org/ncov/asia) (11). Phylogenetic analysis of this virus genome showed that it was grouped in SARS-CoV-2 clade A2a. The genome contained two mutations not observed in the closest previously observed genotype, which occurred in samples from Ireland, Italy, Greece, Hungary, the Czech Republic, Denmark, the United States, Turkey, Jordan, Russia, Georgia, Vietnam, Argentina, Switzerland, Spain, New Zealand, India, Taiwan, Singapore, and Japan (Fig. 1). Because of the wide geographic spread of that closest ancestral genotype, it is difficult to infer the specific route by which the strain came to Bangladesh. As more SARS-CoV-2 genomes from Bangladesh and around the world are sequenced, we will be able to better monitor new introductions into and transmission dynamics within Bangladesh.

In total, 9 mutations were observed in CHRF_nCoV19_0001, compared to the reference genome for the Wuhan strain (GenBank accession no. MN908947.3) from December 2019 (Table 1). These mutations included the spike protein D614G mutation that is enriched in recent SARS-CoV-2 isolates, especially from Europe and North America (12). Other mutations of interest included the position 28881 to 28883 GGG→AAC mutation, which changes the sequence of nucleoprotein N at positions 203 and 204 from RG to KR. In the Nextstrain global build of 15 May 2020, this mutation is predicted to have emerged at the end of January 2020 and is present in many isolates from clade A2a (11). Finally, one mutation observed is unique to CHRF_nCoV19_0001, i.e., A1163T. This mutation leads to a I300F change in open reading frame 1a (ORF1a) (on the nsp2 protein), and its prevalence in Bangladesh should be monitored closely.

**TABLE 1:** Mutations present in CHRF_nCoV19_0001 in relation to the ancestral Wuhan strain (GenBank accession no. MN908947.3)

Nucleotide position	Reference nucleotide	Mutated nucleotide	Amino acid change	Comments
241	C	T	Noncoding	
1163	A	T	ORF1a, I300F	nsp2, function unclear
3037	C	T	No change	
14408	C	T	ORF1b, P314L	nsp13, RNA-dependent RNA polymerase
17019	G	T	ORF1b, E1184D	nsp14, helicase
23403	A	G	S, D614G	S (spike protein), mutation may affect transmission/virulence
28881	G	A	N, R203K	N, involved in RNA packaging
28882	G	A	No change	
28883	G	C	N, G204R	N, involved in RNA packaging

We are currently sequencing more genomes from different regions of Bangladesh and from patients with different clinical features to further investigate the spread of COVID-19 and to monitor the evolution of SARS-CoV-2 in Bangladesh.

### Data availability.

The SARS-CoV-2 genome from Bangladesh was deposited in the GISAID database (accession no. EPI_ISL_437912) and GenBank (accession no. MT476385). The raw reads have been deposited in the NCBI Sequence Read Archive (SRA accession no. SRR11801823). The BioProject and BioSample accession no. are PRJNA633241 and SAMN14938301, respectively.
